# Supplementary material for: Content-rich biological network constructed by mining PubMed abstracts
Source: BMC Bioinformatics. 2004 Oct 8;5:147. doi: 10.1186/1471-2105-5-147 (PMC528731; doi:10.1186/1471-2105-5-147)
Supplement: Additional File 2 — The original results of the above study (non-essential files are deleted to keep the file size under the limit set by BMC bioinformatics). [file 1471-2105-5-147-S2.bz2 › chilibotAdditionalFile2/dip05/607ID9786864E10989/html/LEP_LEPR.html]

 


 **LEP** and **LEPR** 
  
Found 5619 abstracts in PubMed, retrieved 05.  
 

 What does Google say? 
 PDF only 
| .edu only 

---

**Interactive relationship** (e.g. stimulation, inhibition, etc)

**Neutral relationship**- CONCLUSIONS Together these data show that the effect of leptin  [ **LEPR** ]   [ **LEP** ]  on LV remodeling is not attributable to weight loss alone, indicating that leptin  [ **LEPR** ]   [ **LEP** ]  has antihypertrophic effects on the heart, either directly or through a leptin  [ **LEPR** ]   [ **LEP** ]  regulated neurohumoral pathway.  Ref: 12885755 CirculationCirculation,

**Non-interactive relationship** (e.g. studied together, co-existance, homology, etc.)

- The highest leptin  [ **LEPR** ]   [ **LEP** ]  levels were found in quintile 1 geometric mean GM 5.42.  Ref: 12885988 Thorax, 2003
- This paper reviews the role of leptin  [ **LEPR** ]   [ **LEP** ]  in the regulation of body weight, as well as different aspects related to obesity.  Ref: 12886336 An Sist Sanit Navar, 1999
- The discovery of leptin  [ **LEPR** ]   [ **LEP** ] , besides causing a revolution in physiological knowledge about the regulation of body weight, awakes an interest in fat tissue as an endocrine organ and its active contribution to the establishment of energy deposits.  Ref: 12886336 An Sist Sanit Navar, 1999
- Leptin  [ **LEPR** ]   [ **LEP** ]  may play a role in the pathogenesis of cardiovascular morbidity and mortality related to impaired lung function.  Ref: 12885988 Thorax, 2003
- Disruption of Leptin  [ **LEPR** ]   [ **LEP** ]  Signaling Contributes to Cardiac Hypertrophy Independently of Body Weight in Mice.  Ref: 12885755 CirculationCirculation, 2003
- Like other cytokines, leptin  [ **LEPR** ]   [ **LEP** ]  is also able to play a role in the complex regulation of diverse metabolic aspects.  Ref: 12886336 An Sist Sanit Navar, 1999
- Much evidence suggests that leptin  [ **LEPR** ]   [ **LEP** ]  plays the same roles in human beings.  Ref: 12884645 Przegl Lek, 2003
- One of the roles of leptin  [ **LEPR** ]   [ **LEP** ]  in animals is regulation of the energy balance by decreasing food intake and increasing energy expenditure.  Ref: 12884645 Przegl Lek, 2003
- CONCLUSION Individuals with impaired lung function have raised serum leptin  [ **LEPR** ]   [ **LEP** ]  levels.  Ref: 12885988 Thorax, 2003
- Impaired lung function and serum leptin  [ **LEPR** ]   [ **LEP** ]  in men and women with normal body weight a population based study.  Ref: 12885988 Thorax, 2003
- Disruption of leptin  [ **LEPR** ]   [ **LEP** ]  signaling may represent a novel mechanism in LVH and related cardiovascular disorders.  Ref: 12885755 CirculationCirculation, 2003
- Since the discovery that the placenta, gastric mucous or the star shaped cells of the liver Kupffer cells, are places that produce leptin  [ **LEPR** ]   [ **LEP** ] , a great range of functions have been attributed to this hormone.  Ref: 12886336 An Sist Sanit Navar, 1999
- Within a prospective cohort study, we measured testosterone, sex hormone binding globulin SHBG and IGF I, IGF binding protein IGFBP 1, IGFBP 3, insulin and leptin  [ **LEPR** ]   [ **LEP** ] , in plasma from 149 cases and 298 controls.  Ref: 12883384 Eur J Cancer Prev, 2003
- Leptin  [ **LEPR** ]   [ **LEP** ]  infusion completely reversed the increase in wall thickness with partial resolution of myocyte hypertrophy.  Ref: 12885755 CirculationCirculation, 2003
- To separate the direct contribution of leptin  [ **LEPR** ]   [ **LEP** ]  deficiency from mechanical effects of obesity, we induced weight loss in 6 to 8 month old ob ob mice either by leptin  [ **LEPR** ]   [ **LEP** ]  infusion or caloric restriction.  Ref: 12885755 CirculationCirculation, 2003
- Leptin  [ **LEPR** ]   [ **LEP** ] , principally secreted by the adipocytes, interacts with hypothalamic receptors, and is capable of activating diverse neuroendocrinal routes responsible for controlling the balance between energy intake and expenditure.  Ref: 12886336 An Sist Sanit Navar, 1999
- The absence of an association between plasma levels of androgens and prostate cancer risk in analytical studies, despite the strong indirect evidence of their tumour stimulating effects, may reflect the complex and mostly inverse associations of androgenicity to IGF I, insulin and leptin  [ **LEPR** ]   [ **LEP** ]  which are hormones that have also been implicated as risk factors for prostate cancer.  Ref: 12883384 Eur J Cancer Prev, 2003
- Compared with quintile 5, the odds of having an increased serum leptin  [ **LEPR** ]   [ **LEP** ]  level in quintiles, 3, and 4 were 2.26 95% confidence interval CI 1.54 to 3.3.  Ref: 12885988 Thorax, 2003
- The use of recombinant leptin  [ **LEPR** ]   [ **LEP** ]  in patients with obesity is mentioned.  Ref: 12884645 Przegl Lek, 2003
- A study was undertaken to determine whether leptin  [ **LEPR** ]   [ **LEP** ] , a hormone that can promote atherothrombosis, is raised in individuals with impaired lung function.  Ref: 12885988 Thorax, 2003
- Dysregulation of leptin  [ **LEPR** ]   [ **LEP** ] , a neurohormone essential to energy homeostasis, is implicated in the pathogenesis of obesity.  Ref: 12885755 CirculationCirculation, 2003
- ... testosterone and SHBG correlated inversely with IGF I, IGFBP 3, insulin, leptin  [ **LEPR** ]   [ **LEP** ]  and body mass index BMI.  Ref: 12883384 Eur J Cancer Prev, 2003
- To further investigate this issue, we studied the interrelationships between androgenicity and insulin like growth factor I IGF I, insulin and leptin  [ **LEPR** ]   [ **LEP** ] .  Ref: 12883384 Eur J Cancer Prev, 2003
- Leptin  [ **LEPR** ]   [ **LEP** ]  and body weight regulation .  Ref: 12886336 An Sist Sanit Navar, 1999
- METHODS AND RESULTS We measured left ventricular LV wall thickness and LV mass with echocardiography in mice lacking leptin  [ **LEPR** ]   [ **LEP** ]  ob ob, n=15 or functional receptor db db, n=10 and controls at, and 6 months of age.  Ref: 12885755 CirculationCirculation, 1999
- METHODS Data from non obese participants in the Third National Health, Nutrition, and Examination Survey n=2808 were analysed to determine the relationship between circulating leptin  [ **LEPR** ]   [ **LEP** ]  levels and forced expiratory volume in 1 second FEV 1 values divided into quintiles quintile 1, FEV 1 predicted <.  Ref: 12885988 Thorax, 2003
- RESULTS Serum leptin  [ **LEPR** ]   [ **LEP** ]  levels changed along the FEV 1 gradient.  Ref: 12885988 Thorax, 2003
- Leptin  [ **LEPR** ]   [ **LEP** ]  is a hormone that is released mainly by adipocytes.  Ref: 12884645 Przegl Lek, 2003
- Indices of free testosterone showed an inverse linear correlation with leptin  [ **LEPR** ]   [ **LEP** ]  P< ; 0.01, and a strong drop in the 5th quintile of BMI.  Ref: 12883384 Eur J Cancer Prev, 2003
- This brief overview focuses on the history of leptin  [ **LEPR** ]   [ **LEP** ]  discovery, regulation of circulating leptin  [ **LEPR** ]   [ **LEP** ]  levels, and physiological role scientifically proved and being under research.  Ref: 12884645 Przegl Lek, 2003
- Because leptin  [ **LEPR** ]   [ **LEP** ]  has cardiovascular bioactivity, we hypothesized that disruption of leptin  [ **LEPR** ]   [ **LEP** ]  signaling mediates the development of obesity associated LVH.  Ref: 12885755 CirculationCirculation, 2003
- Besides, leptin  [ **LEPR** ]   [ **LEP** ]  participates es in the immune and inflammatory responses, in reproduction, in processes related to angiogenesis, control of arterial pressure, lipolysis, etc.  Ref: 12886336 An Sist Sanit Navar, 1999
- Interrelationships between plasma testosterone, SHBG, IGF I, insulin and leptin  [ **LEPR** ]   [ **LEP** ]  in prostate cancer cases and controls.  Ref: 12883384 Eur J Cancer Prev, 2003
